# Medically important bacteria isolated from commercial herbal medicines in Kampala city indicate the need to enhance safety frameworks

**DOI:** 10.1038/s41598-022-21065-y

**Published:** 2022-10-05

**Authors:** Abdul Walusansa, Jesca. L. Nakavuma, Savina Asiimwe, Jamilu. E. Ssenku, Dickson Aruhomukama, Tahalu Sekulima, Hussein. M. Kafeero, Godwin Anywar, Esther Katuura, Alice Nabatanzi, Nathan. L. Musisi, Arthur. K. Tugume, Esezah. K. Kakudidi

**Affiliations:** 1 Department of Plant Sciences, Microbiology and Biotechnology, School of Biosciences, Makerere University, Kampala, Uganda; 2 Department of Medical Microbiology and Immunology, Faculty of Health Sciences, Habib Medical School, Islamic University in Uganda, Kampala, Uganda; 3 Department of Biomolecular and Biolaboratory Sciences, College of Veterinary Medicine, Animal Resources and Biosecurity, Makerere University, Kampala, Uganda; 4 Department of Medical Microbiology and Immunology, Faculty of Health Sciences, Busitema University, Mbale, Uganda; 5 Department of Immunology and Molecular Biology, College of Health Sciences, Makerere University, Kampala, Uganda

**Keywords:** Biochemistry, Microbiology, Molecular medicine

## Abstract

The high global bacterial infection burden has created need to investigate the neglected potential drivers of pathogenic bacteria, to inform disease prevention. Kampala is facing a proliferation of herbalists, selling herbal medicine (HM), of largely unregulated microbiological quality. We evaluated the bacterial contamination burden in HM sold in Kampala, to support evidence-based redress. The total viable loads (TVL), total coliform counts (TCC), *E. coli* counts, and prevalence of selected bacterial strains in 140 HM were examined using conventional culture, following the guidelines of World Health Organization (WHO), and Uganda National Drug Authority (NDA). Data were analyzed using D'Agostino-Pearson test, frequencies, proportions, Chi-square, and Mann–Whitney U test with STATA version-15.0. Fifty (35.7%), fifty-nine (42.1%), and twelve (8.6%) HM were unsafe for human use because they exceeded WHO’s permissible limits for TVL, TCC, and *E. coli* counts respectively. Solids had significantly higher mean TVL than liquids. Violation of NDA’s guidelines was significantly associated with high TVL. Fifty-nine bacteria, viz., *Klebsiella pneumoniae* (n = 34; 57.6%), *Escherichia. coli* (12; 20.3%), *Staphylococcus aureus* (7; 11.9%), *Klebsiella oxytoca* (3; 5.1%), *Bacillus cereus*, *Pseudomonas aeruginosa*, and *Enterobacter* spp. (1; 1.7% each), were isolated from 45 (32.1%) samples. These bacteria can cause severe clinical diseases, and promote deterioration of HM potency.

## Introduction

Infectious diseases are among the major causes of death and hospitalization worldwide, and those caused by bacteria are more common in some parts of Africa including Uganda^1^. In Eastern Africa, the burden incurred by bacterial pathogens manifests more in form of diarrheal and/or respiratory diseases. For example, in Uganda and Tanzania, diarrheal and respiratory ailments of bacterial origin are ranked among the six major causes of both adult and childhood mortality^2^. Since the leading factors in escalating these infections may vary from one setting to another^3^, the establishment of community- or area specific research and/or other interventions is necessary for effective thwarting of the burden. In Uganda, like some other parts of the world, the interventions aimed at counteracting infectious disease spread are somewhat inclined towards the human-to-human and/or human-to-animal linkages^4^. Though neglected to some extent, the potential role some plant resources may play in the community spread of bacterial pathogens could be substantial. Such plant materials are not only limited to food but also herbal medicines (HM)^1,5–7^.

In Uganda, like most other parts of the world, HM trade has been increasing in a few past decades^8^. Of recent, the ratio of traditional medical practitioners (TMP) to the general population in Uganda was reported to be between 1:200 and 1:400, contrasting greatly with the ratio of formal health personnel to the general population which was ≤ 1:20,000 (WHO^9,10^). The herbalists also offer consultation services, and/or sell HM for managing common ailments. The evolution of microbial resistance to conventional drugs, and the affordability and accessibility of HM have been reported as some of the key factors promoting the ongoing increase in herbal medicine use, especially in the developing world^9,10^. Kampala being Uganda’s major capital and commercial center, has a greater number of herbal medicine business ventures compared to other cities in the country^5^. This could partly be attributed to the high demand, and the lucrative market offered by the large population of residents, travelers, and the business community in the city^11^. The resident population in Kampala city mostly comprises of low-income earners that live in the suburbs, and have high inclination to HM use^5,12–14^.

In the recent past, the population in Kampala city has been experiencing persistent outbreaks of bacterial diseases such as typhoid fever and *E. coli* infection among others^15,16^. In this city, HM are widely popularized by the herbalists as being absolutely safe for human use. However, the microbiological quality of these HM is not routinely monitored. Besides, adverse effects of HM have been reported to be potentially linked to bacterial contamination in some parts of the world^1,6,7^. Such bacteria may include *Streptococcus pneumoniae*, *Salmonella* spp., *Escherichia coli*, *Pseudomonas aeruginosa*, *Staphylococcus aureus*, and *Klebsiella pneumoniae*, among others^7,17^. Several strains belonging to some of the species which may contaminate HM, such as *E. coli*, *Salmonella* spp., and *Streptococcus pneumoniae* have been implicated in severe diarrheal and/or respiratory diseases^1^. Further, some bacterial species, particularly members of the family Enterobacteriaceae (the coliforms), like *E. coli* have been specified by the World Health Organization (WHO), as indicators of fecal contamination^18^. Therefore, the existence of *E. coli* in HM does not only indicate gross violation of hygiene standards, but also the potential presence of other, more virulent, enteric pathogens such as *Salmonella* spp. and *Shigella* spp. among others^5,19^. Information about the degree of bacterial loading, and the profiles of bacteria that inhabit HM in particular settings is essential in improving the mitigation strategies.

The aim of this study therefore, was to isolate and characterize selected bacteria that could be associated with diarrhea and/or respiratory infections such as *Salmonella* spp., *Shigella*, *B. cereus*, *K. pneumoniae*, *S. aureus* and *E. coli* from commercial HM sold in Kampala city. The overall degree of bacterial contamination was also assessed by using standard microbiological parameters such as the Total Coliform Counts (TCC), Total Viable Counts (TVL), and *E. coli* counts. The findings could contribute to the attainment of evidence-informed public health policy and practice.

## Results

### Socio-demographic profile of participants

Majority (n = 36, 55.4%) of the respondents were men, hence females were 44.6% (n = 29). Most of the participants were in the youthful age category (n = 24, 36.9%), followed by the middle aged (n = 39, 60%), while only two (3.1%) were elderly. Most of the participants (n = 26, 40.0%) had attended secondary education, while very few (3, 4.6%) had attained tertiary education. The majority of the respondents had practiced commercial traditional medicine for a duration of 5–15 years, and these constituted 72.3% (n = 47). Most participants (39, 60.0%), earned a net monthly profit of UGX 730,000 ($ 200) ≤ 1,460,000 ($ 400), while the minority (5, 7.7%) earned above UGX 1,825,000 ($ 500) from HM sales (Table 1).

**Table 1:** Socio-demographic characteristics of commercial herbalists in Kampala city (N-65).

Variables	Frequency**,** n (%)
**Gender**	
Male	36 (55.4)
Female	29 (44.6)
**Age (years)**	
18–24(youths)	24 (36.9)
25–63 (middle aged)	39 (60.0)
≥ 64 (elderly)	2 (3.1)
**Nationality**	
Ugandan	65 (100)
Non-Ugandan	0 (0.0)
**Marital status**	
Married	39 (60.0)
Single	26 (40.0)
**Education**	
None	8 (12.3)
Primary	26 (40.0)
Secondary	28 (43.1)
Tertiary	3 (4.6)
**Years of experience in HM**	
5 ≤ 15	47 (72.3)
16 ≤ 20	15 (23.1)
> 20	3 (4.6)
**Type of HM establishment**	
Market stalls	18 (27.7)
Herbal shops	17 (26.2)
Road side and/or mobile stalls	30 (46.1)
**Estimated monthly net profit from HM, UGX (USD)**	
< 730,000 (200)	9 (13.8)
730,000 ≤ 1,460,000 (400)	39 (60.0)
1,460,000 < 1,825,000 (500)	12 (18.5)
≥ 1,825,000 (500)	5 (7.7)

*UGX* Uganda Shillings, *$* United States Dollar, *HM* Herbal Medicine.

### Characteristics of the herbal medicines from which bacterial contaminants were isolated

**Figure 1:**
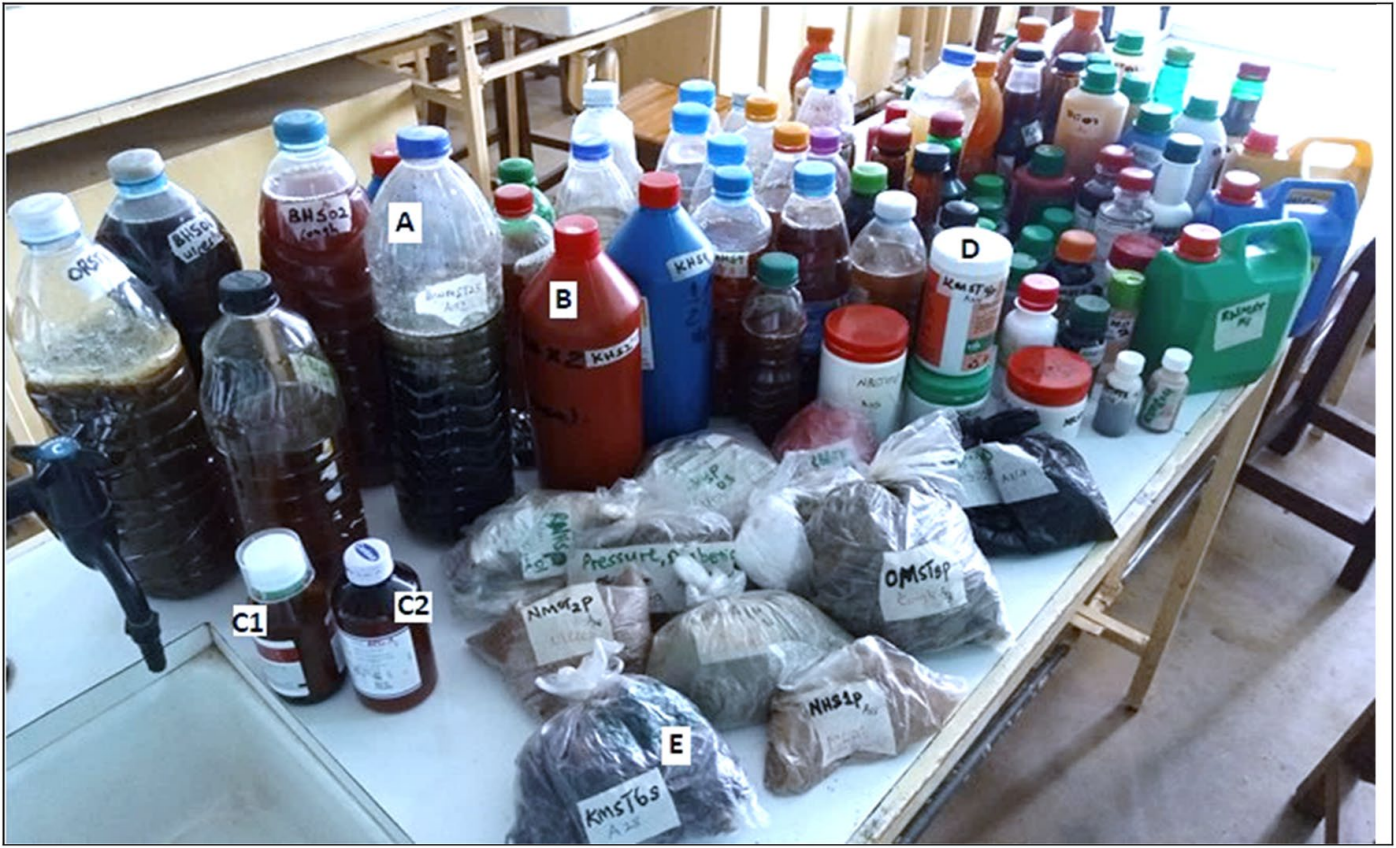
Some of the herbal medicine samples from which contaminant bacteria were isolated. The samples included both solids and liquids.

Out of the 140 herbal medicine (HM) samples, 114 were liquids while 26 were solids. In addition, two conventional antibiotic drugs were included as controls. The HM samples were availed on market in different packaging materials namely; bottles (116; 82.6%), polyethene (12; 8.6%), Tins (6; 4.3%), papers (5; 3.6%), and bare (unwrapped) (1; 0.9%). Figure 1 below shows some of the samples that were investigated in this research. Most of the HM (87; 62.1%), were packaged in original material (without evidence of package recycling), the re-used packages were (53; 37.83%), while the bare/unwrapped was (1, 0.07%). One hundred and one (101, 72.1%) samples were visibly clean, while 39 (27.9%) appeared dusty. Though most (77, 55.0%) samples satisfied at least (4 of the10) conditions enshrined in herbal medicine packaging and branding guidelines provided by Uganda National Drug Authority (NDA)^20^, 63 samples (63, 45.0%) were in absolute disconformity (did not satisfy any of the 10 conditions), while none of the samples (0, 0%) conformed to all the 10 conditions. The ailments suggested to be treated by these HM belonged to 12 disease categories; gastro-intestinal tract infections being the largest category (constituting 14 diseases), followed by respiratory infections (10 diseases), and urinogenital and reproductive disorders (8 diseases); the smallest categories were the metabolic disorders and bone diseases with one ailment each (Table 2).

**Table 2:** Ailments that were suggested to be treated by the herbal medicines from which bacterial contaminants were isolated in this study.

Disease category {N}	Diseases {F}
Gastro-intestinal Tract Infections {14}	Diarrhea {51}, typhoid {30}, heartburn {1}, constipation {1}, appetite loss {4}, obesity {1}, halitosis (bas smelling mouth) {4}, abdominal hernia {6}, bleeding gun {4}, peptic ulcers {21}, vomiting {7}, mouth sores {11}, brucellosis; {1}, tooth decay {1}
Respiratory infections {10}	Cough {77}, asthma {13}, flu {24}, pneumonia {4}, tuberculosis (TB) {2}, sinuses {16}, abnormal breath {1}, bronchitis {1}, common cold {3}, rhinitis {1}
Urinogenital, and reproductive disorders {8}	Erectile dysfunction {22}, Genital warts {2}, Syphilis {11}, uterine fibroids {7}, menstrual imbalances {1}, infertility in women {1}, vaginal candidiasis {8}, kidney diseases {5}
Aches {5}	Headache {9}, back ache {1}, tooth ache {4}, chest pain {3}, abdominal {10}
Cardiovascular diseases {4}	Hypertension {44}, Anemia {1}, heart diseases {2}, atherosclerosis (thickening/hardening of the arteries) {1}
Skin Infections {4}	Pimples {5}, bromadrosis (smelly feet) {1}, bromhidrosis (excessive unpleasant odor from the skin) {1}, measles {10}
Ear, Nose and Throat {4}	Nose bleeding {3}, sore throat {2}, tonsillitis (inflammation of the tonsils) {5}, pus in ears {4}
Cancers {3}	Breast cancer {1}, colon cancer {1}, cancer in general {1}
Optical Complications {2}	Itchy eyes {2}, impaired vision {2}
Metabolic Disorders {1}	Diabetes {40}
Bone diseases {1}	Osteoporosis (weak, brittle bones) {1}
Other complications {9}	HIV/AIDS {1}, Malaria {11}, low immunity {2}, yellow fever {2}, allergy {8}, fever {15}, paralysis {6}, general body weakness {6}, ETC {5}

*N* number of diseases in a disease category, *F* frequency, *HIV/AIDS* human immunodeficiency virus/acquired immunodeficiency syndrome, *ETC* Et cetera/and the rest (unspecified diseases).

### Total viable loads (TVL) of bacteria isolated from selected herbal medicines sold in Kampala City

**Figure 2:**
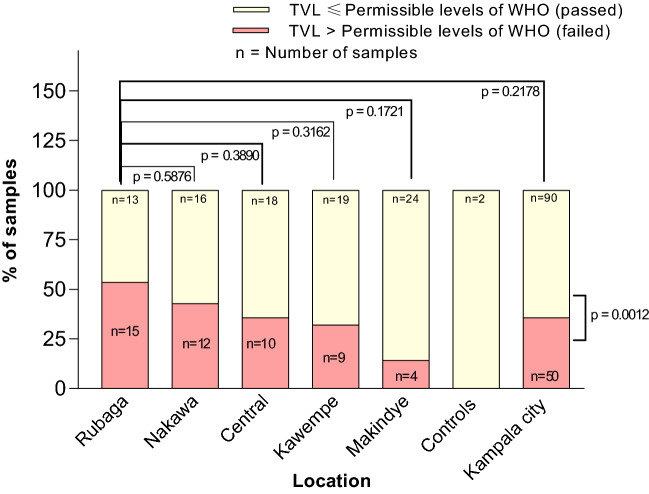
Conformity of the herbal medicines sold in Kampala to the permissible bacterial Total Viable Loads (TVL), that are recommended by World Health Organization (WHO)^18^. The p values show the significancy of differences in the proportions of samples that possessed unacceptable TVL.

Out of the 140 samples screened, 50 (35.7%) possessed total viable loads (TVL) of bacteria that exceeded the maximum acceptable levels (10^5^ CFU/ml for liquids and 10^7^ CFU/g for solids), that are recommended by the World Health Organization (WHO)^18,19^. However, the samples that exceeded permissible limits were significantly fewer than those that passed (*χ*^2^, *p* = 0.0012). Though most (15 out of 50), samples which did not conform to the WHO standards were found in Rubaga, a Chi-square test revealed that such HM did not significantly differ from those traded in the other divisions of the city (*p* ≤ 0.05) (Fig. 2).

**Figure 3:**
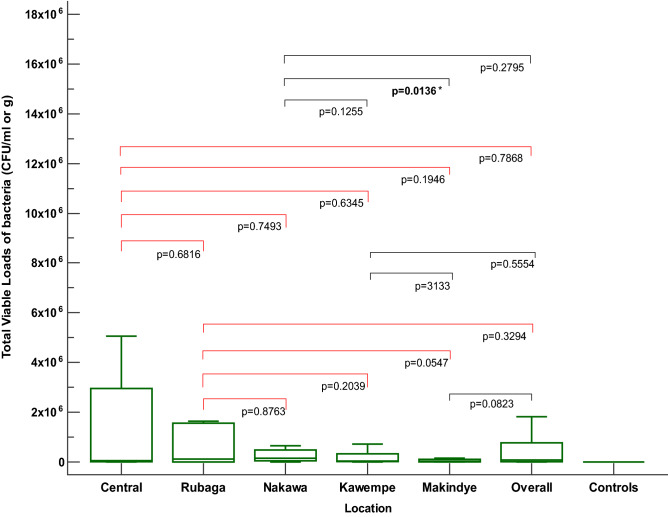
Distribution of total viable loads (TVL) of bacteria in herbal medicines sold in Kampala. The p values show the significance of differences in TVL in the five divisions of the city, viz; Kampala central, Rubaga, Nakawa, Kawempe, and Makindye (p < 0.05).

The mean TVL from all the 140 samples (solids and liquids) was 126.407 × 10^4^ CFU/ml or g, and it exceeded the WHO’s permissible levels. The mean TVL values were (286.334, 125.919, 77.351, 52.789, and 35.821) × 10^4^ CFU/ml or g for Rubaga, Kampala central, Nakawa, Makindye, and Kawempe divisions, respectively. There were no significant variations in the mean TVL of the five divisions, as well as the overall mean, except for Nakawa and Makindye (Mann–Whitney U; *Z* = 2.467, *p* = 0.0136) (Fig. 3).

For the liquid HM, the lowest TVL was 0.000 × 10^4^ CFU/ml (recorded in 14 samples), while the highest was 1420.000 × 10^4^ CFU/ml. For the solid HM, the highest was TVL was 1670.000 × 10^4^ CFU/g and the least was 0.180 × 10^4^ CFU/g. (Table 3). Overall, the highest TVL was 1670.000 × 10^4^ CFU/g (observed in one solid sample), while the lowest was 0.000 × 10^4^ CFU/ml (recorded in 14 liquid samples). Consequently, the mean TVL of the solid samples (215.660 × 10^4^ CFU/g) was significantly higher than that (106.051 × 10^4^ CFU/ml) in liquids (Mann–Whitney U; *Z* = − 2.136, *p* = 0.0326). Similarly, the mean TVL was significantly higher in solids than liquids in Makindye (Z = − 2.135, *p* = 0.0327). On the contrary, the mean TVL was significantly higher in liquids than the solid samples in Rubaga division (Z = − 2.673, *p* = 0.0075), while there was no significant variation in the mean TVL of solids and liquids in Kawempe, Kampala central, and Nakawa divisions (Table 3).

**Table 3:** Total viable loads (TVL) of bacterial isolated from selected herbal medicines sold in Kampala City.

Variable	n	Min. TVL × 10^4^ (CFU/ml/g)	Max. TVL × 10^4^ (CFU/ml/g)	Mean ± SD × 10^4^ (CFU/ml/g)	Test statistic	p value	95% CI × 10^4^
**Kawempe division**							
Liquid	23	0.000	844.000	42.230 ± 113.496			
Solid	5	0.180	16.800	6.342 ± 6.294	Z = − 0.150	0.8808	− 71.820 to 4.630
**Outlet type**							
Roadside/mobile stalls	14	0.000	528.000	61.215 ± 143.823 REF			
Herbal shops	7	0.040	44.000	14.637 ± 15.505	W = 49.00	1.0000	− 6.710 to 43.920
Market stalls	7	0.180	16.000	6.219 ± 5.572	Z = − 0.485	0.6276	− 7.400 to 74.500
**Packaging materials**							
Recycled	5	16.000	528.000	124.860 ± 225.661			
Original	22	0.000	200.000	16.450 ± 41.911	Z = 0.343	0.7313	− 85.970 to 11.480
None/bare	1	6.730	6.730	6.730 ± (NA)	NA	NA	NA
**Hygiene appearance**							
Dusty	11	0.030	528.000	58.031 ± 156.025			
Clean	17	0.000	200.000	21.451 ± 47.737	Z = − 0.706	0.4804	− 3.810 to 16.620
**Kampala central**							
Liquid	22	0.000	506.000	150.450 ± 190.376			
Solid	6	1.000	143.000	38.750 ± 52.788	Z = 0.112	0.9107	− 315.150 to 29.140
**Outlet type**							
Herbal shops	9	0.000	452.000	146.097 ± 192.960 REF			
Market stalls	11	0.000	387.000	105.650 ± 169.696	t = − 0.499	0.6240	− 0.021 to 0.013
Roadside or mobile stalls	8	0.860	506.000	133.171 ± 184.942	t = − 0.141	0.8901	− 0.021 to 0.018
**Packaging materials**							
Recycled	10	0.310	506.000	281.331 ± 190.846			
Original	18	0.000	344.000	39.580 ± 88.853	Z = 3.169	0.0015*	− 386.660 to − 33.150
None/bare	0	NA	NA	NA			
**Hygiene status**							
Clean	21	0.000	452.000	126.977 ± 171.119			
Dusty	7	0.000	506.000	122.746 ± 204.937	Z = 0.558	0.5769	− 137.090 to 43.000
**Makindye division**							
Solid	5	0.000	668.000	184.702 ± 291.620			
Liquid	23	0.000	212.000	23.226 ± 57.545	Z = − 2.135	0.0327*	0.600 to 254.000
**Outlet type**							
Roadside or mobile stalls	8	0.000	668.000	152.330 ± 232.002 (REF)			
Herbal shops	10	0.000	182.000	21.090 ± 56.666	Z = 0.756	0.4499	− 247.370 to 1.990
Market stalls	10	0.000	15.200	4.856 ± 5.017	Z = 0.938	0.3483	− 245.900 to 3.380
**Packaging materials**							
Recycled	15	0.000	668.000	96.027 ± 180.783			
Original	13	0.000	15.400	2.900 ± 4.472	Z = 2.425	0.0153*	− 82.000 to − 0.970
None/bare	0	NA	NA	NA			
**Hygiene appearance**							
Clean	22	0.000	668.000	62.24 ± 154.193			
Dusty	6	0.000	82.000	18.137 ± 31.825	Z = − 0.112	0.9106	− 6.680 to 12.280
**Rubaga division**							
Liquid	23	0.000	1420.000	159.667 ± 373.599			
Solid	5	27.000	1670.000	869.000 ± 739.85	Z = − 2.673	0.0075*	26.970 to 1369.970
**Outlet type**							
Market stalls	8	0.000	1370.000	395.751 ± 562.304 (REF)			
Herbal shops	12	0.000	1420.000	250.132 ± 486.239	Z = 0.193	0.8468	− 711.970 to 122.800
Roadside or mobile stalls	8	0.000	1670.000	231.220 ± 582.716	Z = 0.7927	0.2630	− 920.000 to 31.900
**Packaging materials**							
Original	19	0.000	1670.000	189.516 ± 482.026			
Recycled	9	0.000	1370.000	490.727 ± 569.029	Z = 0.1267	0.1267	− 5.560 to 1008.100
None/bare	0	NA	NA	NA			
**Hygiene status**							
Dusty	7	0.000	1670.000	964.286 ± 651.431			
Clean	23	0.000	712.000	60.350 ± 156.649	Z = − 2.762	0.0058*	NA
**Nakawa division**							
Liquid	23	0.070	536.000	91.093 ± 160.606			
Solid	5	9.880	16.800	14.136 ± 2.772	Z = 0.210	0.8337	− 103.200 to 11.530
**Outlet type**							
Market stalls	9	0.080	536.000	105.193 ± 193.247 (REF)			
Herbal shops	11	2.570	488.000	91.939 ± 158.668	W = 80.000	0.2947	− 42.300 to 39.790
Roadside or mobile stalls	8	0.070	120.000	25.970 ± 39.420	W = 80.000	0.9626	− 208.000 to 20.190
**Packaging materials**							
Recycled	14	0.080	536.000	141.776 ± 190.909			
Original	14	0.070	40.000	12.926 ± 11.797	t = − 2.521	0.0182*	− 0.023 to − 0.002
None/bare	0	NA	NA	NA			
**Hygiene appearance**							
Clean	20	0.070	536.000	62.962 ± 132.167			
Dusty	8	0.080	488.000	113.324 ± 187.251	W = 282.000	0.7086	− 15.870 to 31.070
**Kampala city**							
Solid	26	0.180	1670.000	215.660 ± 460.046 (REF)			
Liquid	114	0.000	1420.000	106.051 ± 22.767	*Z* = − 2.136	0.0326*	0.180 to 13.880
Controls	2	0.000	0.0006	0.0003 ± 0.0004	*Z* = − 1.975	0.0483 *	NA
**Outlet type**							
Market stalls	45	0.000	1370.000	128.676 ± 289.032 (REF)			
Herbal shops	49	0.000	1420.000	141.654 ± 291.734	Z = 0.708	0.4788	− 8.190 to 1.880
Roadside or mobile stalls	46	0.000	1670.000	118.597 ± 284.286	Z = − 0.052	0.9584	− 6.540 to 4.400
Controls	2	0.000	0.0006	0.0003 ± 0.0004	Z = − 2.015	0.0439*	NA
**Packaging materials**							
Recycled	53	0.000	1370.000	218.718 ± 322.621 (REF)			
Original	87	0.000	1670.000	70.496 ± 247.317	Z = − 4.159	< 0.0001*	4.800 to 81.950
None/bare	1	6.730	6.730	6.730 ± (NA)	NA	NA	NA
Controls	2	0.000	0.0006	0.0003 ± 0.0004	Z = − 2.192	0.0284*	NA
**Hygiene appearance**							
Dusty	39	0.000	1670.000	238.809 ± 455.331 (REF)			
Clean	101	0.000	844.000	83.004 ± 168.546	Z = − 0.693	0.4883	− 1.710 to 9.880
Controls	2	0.000	0.0006	0.0003 ± 0.0004	Z = 1.909	0.0562	NA
**Conformity to NDA-LP (x/10)**							
0 ≤ NDA-LP ≤ 5	70	0.000	1670.000	189.188 ± 365.875 (REF)			
5 < NDA-LP ≤ 10	70	0.000	844.000	63.626 ± 152.928	Z = 3.444	0.0006*	− 15.400 to − 1.720
Controls	2	0.000	0.0006	0.0003 ± 0.0004	Z = 2.296	0.0217*	NA
**Price (P) (UGX)**							
1,000 ≤ 5,000	83	0.000	1670.000	138.967 ± 314.104 (REF)			
5,000 < P ≤ 10,000	42	0.000	844.000	134.622 ± 201.689	Z = − 1.413	0.1577	− 0.310 to 33.270
> 10,000	15	0.000	182.000	33.906 ± 59.312	Z = 0.430	0.6675	− 7.450 to 4.350
Controls	2	0.000	0.0006	0.0003 ± 0.0004	Z = 2.107	0.0351*	NA
**Qty (mls or grams)**							
10—500	95	0.000	1670.000	92.995 ± 249.640 **(**REF)			
500 < Qty ≤ 1000	38	0.000	1420.000	229.877 ± 365.603	Z = − 2.188	0.0287*	0.180 to 65.000
> 1000	7	0.020	56.400	18.153 ± 24.301	Z = 0.305	0.7607	− 16.370 to 12.420
Controls	2	0.000	0.0006	0.0003 ± 0.0004	Z = 2.032	0.0421*	NA
Overall	140	0.000	1670.000	126.407 ± 286.409	Z = − 0.814	0.4157	− 0.690 to 3.010

*N* number of samples, *Min* minimum, *Max* maximum, *CFU* colony forming units, *ml* milliliter, *g* gram, *NA* not applicable, *Solid* powders and other nonliquids, *Qty* quantity, *NDA-LP* Uganda National Drug Authority Labeling and Packaging guidelines, *x/10* conformity of the sample to NDA-LP scored out of ten.

*P values were significant (< 0.05).

The samples that were sold in recycled packaging materials possessed significantly higher mean TVL than those packed in nonrecycled packages except in Kawempe (Z = 0.343, *p* = 0.7313), and Rubaga divisions (Z = 0.1267, *p* = 0.1267). Both the Mann–Whitney U test and the t-test revealed no significant differences in the mean TVL values among the types of establishments where they were procured (market stalls, herbal shops, roadside and/or mobile stalls) (p < 0.05). The herbal medicines that scored ≤ 5 out of 10 (≤ 50%) in regard to conformity to NDA’s herbal medicine packaging and labeling guidelines possessed significantly higher TVL than those with > 50% (Table 3). Herbal medicines sold at the lower prices (UGX 1000 ≤ 5000) contained greater mean TVL compared to those with high prices (> UGX 5000) but the differences were not significant (Table 3).

### Total coliform counts (TCC), and *E. coli* counts

Fifty nine samples (42.1%) harbored total coliform counts that exceeded the World Health Organization’s maximum permissible limit of ≤ 10^3^ CFU/ml or g^18,19^. Although the majority if these unsuitable samples originated from Kampala central division (n = 17), the proportions were not significantly different from those procured from the rest of the city divisions, as well as the overall TCC (*χ*^2^, *p* < 0.05) (Table 4). The mean TCC was 35.5651 × 10^4^ CFU/ml or g (95% CI = 18.7993 × 10^4^ to 52.3308 × 10^4^ CFU/ml or g), while the range was 624.0000 × 10^4^ CFU/ml or g. The maximum TCC was 624.0000 × 10^4^ CFU/ml. The minimum value was 0.0000 × 10^4^ CFU/ml or g, observed in 64 (45.7%), samples (5 solids and 59 solids). Twelve (8.6%) out of all the sample (N = 140), possessed *E. coli* counts which exceeded the World Health Organization’s maximum permissible limit of ≤ 10 CFU/ml or g^18,19^. Rubaga and Kawempe harbored the greatest number of samples with unacceptable loads of *E. coli* and these were not significantly different from the other divisions of the city except Rubaga (*χ*^2^, *p* < 0.05), which did not exhibit any positive growth of *E. coli* (Table 4).

**Table 4:** Conformity of the commercial herbal medicines in Kampala to the permissible total coliform counts of bacteria recommended by the World Health Organization ^18,19^.

Location	Samples with TCC ≥ WHO permissible limit; No, %	*χ*^2^	p value	95% CI (%)	Samples with *E. coli* count ≥ WHO permissible limit; No, %	*χ*^2^	p value	95% CI (%)
Central (n = 28)	17 (60.7) REF				3 (10.7)			
Nakawa (n = 28)	13 (46.4)	0.587	0.4435	− 19.44 to 44.2	4 (14.3) REF	1.0171	1.8959	− 51.91 to 52.22
Kawempe (n = 28)	11 (39.3)	1.182	0.2769	− 14.68 to 50.7	4 (14.3)	0.0000	1.0000	− 49.24 to 49.24
Rubaga (n = 28)	10 (35.7)	0.1517	0.2181	− 14.68 to 50.7	0 (0.0)	NA	NA	NA
Makindye (n = 28)	8 (28.6)	2.153	0.1423	− 8.64 to 59.54	1 (3.6)	0.0757	0.7832	− 67.32 to 58.89
Overall (n = 140)	59 (42.1)	1.812	0.1782	− 7.7 to 41.01	12 (8.6)	1.101	0.7503	− 67.32 to 58.89
Controls (n = 2)	0 (0.0)	NA	NA	NA	0 (0.0)	NA	NA	NA

*No* number, *TCC* total coliform count, *WHO* World Health Organization, *REF* reference value, *NA* not applicable, *χ*^2^ Chi-square, *CI* confidence interval.

### Prevalence of *K. pneumoniae*, *E. coli*, *S. aureus*, *P. aeruginosa*, *K. oxytoca*, *Enterobacter* spp., *Bacillus cereus*, *Salmonella* spp., and *Shigella* spp. in selected herbal medicines sold in Kampala city

**Figure 4:**
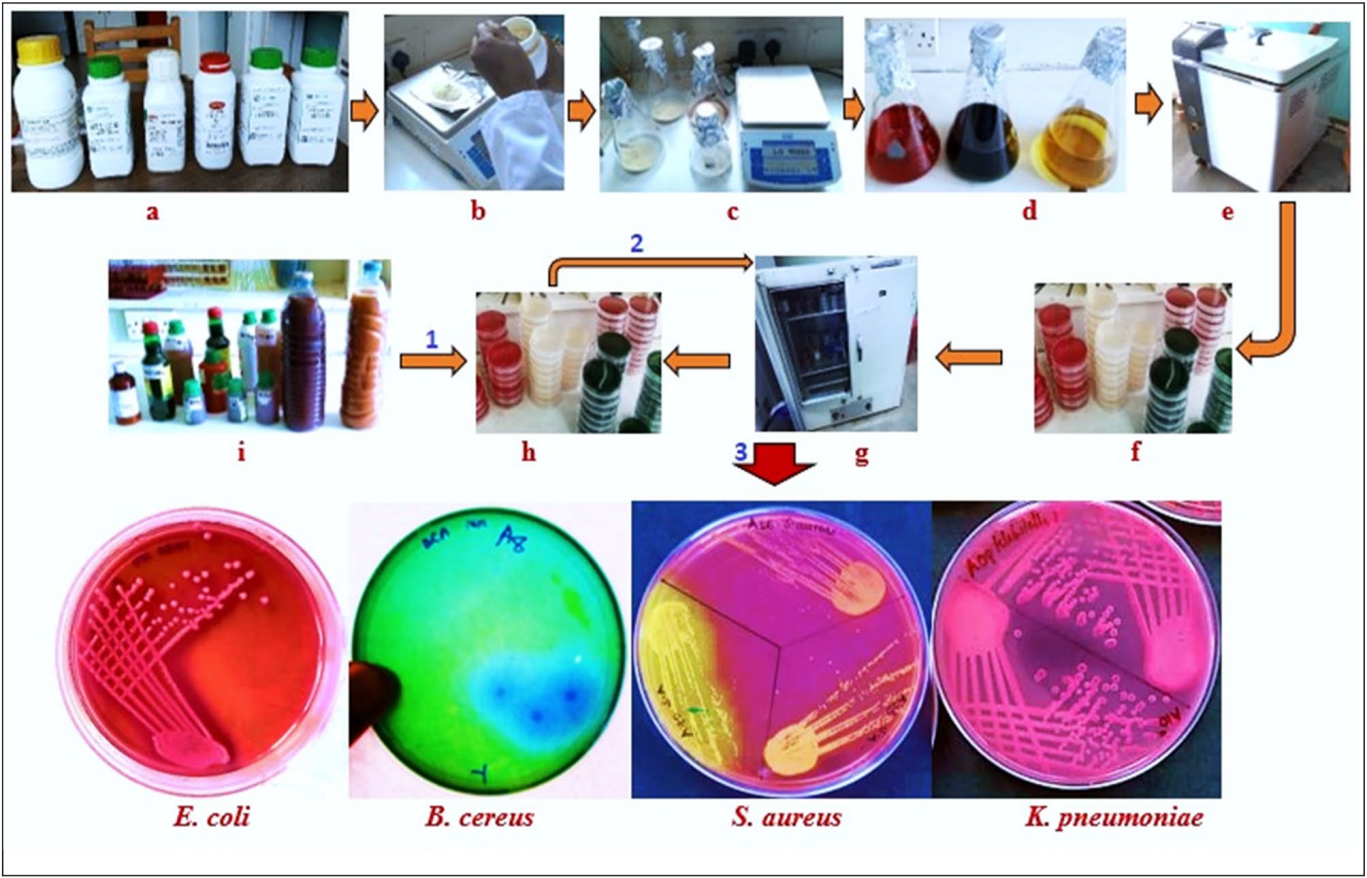
Schematic diagram for isolation of selected bacteria from herbal medicines sold in Kampala city. (**a**) The panel of bacterial culture media used, (**b**) culture media weighed (according to the manufacturer’s instructions) using an electric balance and (**c**), dispensed in conical flasks, then (**d**), dissolved in distilled water followed by autoclaving (**e**). (**f**) Media casted in petri dishes, and (**f**) incubated at 37 °C for 24 h (quality control). (**h**) Media plates that passed the quality test. (**i**) Representative herbal medicine (HM) samples. 1: Inoculation of culture plates with serially diluted HM samples. 2: Incubation of the inoculated culture plates, and 3: Appearance of pure colonies of bacteria on some different media, viz; *E. coli* (hot pink colonies on VRBA), *B. cereus* (bluish colonies on *B. cereus* agar), *S. aureus* (yellow and golden yellow colonies on Mannitol Salt Agar), *K. pneumoniae* (Mucoid pink colonies on MacConkey agar).

Representative cultures for some of the bacteria that grew are shown in Fig. 4. *Pseudomonas aeruginosa*, on Kings’ agar, demonstrated small to medium sized, spindle/irregularly shaped, flat, greenish colonies. *Staphylococcus aureus* on MSA manifested as small, round, raised, smooth, yellow colonies (Fig. 4). On BCA media, *Bacillus cereus* showed medium to large sized, bluish (peacock blue), flat colonies with serrated margins. *E. coli* on VRBA demonstrated hot pink, dry, flat/umbonate colonies, while *Enterobacter* spp. showed mucoid pink to pinkish red colonies, and *Klebsiella* spp., mucoid, golden- or dull-yellow colonies. *K. pneumoniae* presented as mucoid pink colonies on MacConkey agar (Fig. 4). On XLD agar, the characteristic slightly rough/dimpled, red colonies with or without black centers were absent for all the samples, indicating the absence of *Salmonella* spp. or *Shigella* spp. respectively.

**Figure 5:**
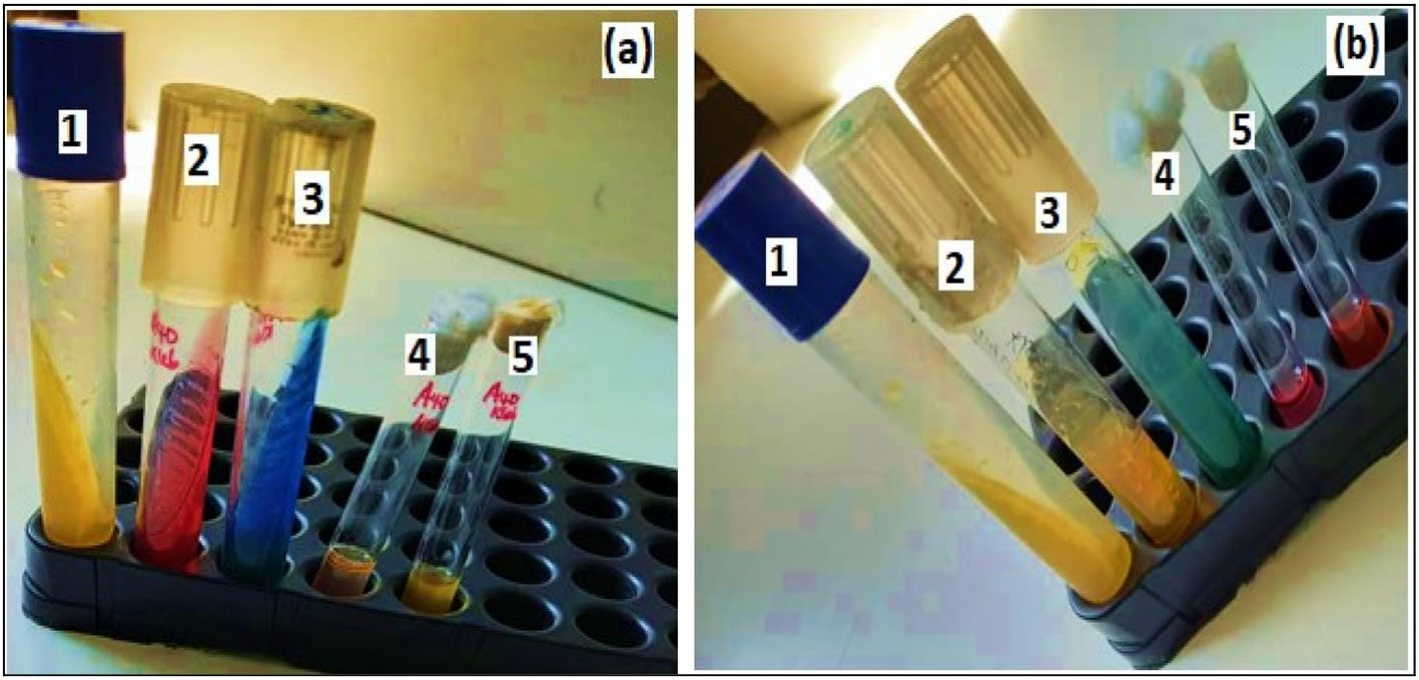
Representative plates for the biochemical phenotypes observed during characterization of bacteria isolated from commercial herbal medicines in Kampala city. (**a**) Panel for *K. pneumoniae*: 1: TSI(A/A), 2: urease (positive reaction)., 3: citrate utilization test (positive reaction), 4: peptone water for Indole (negative reaction), 5: methyl red test (negative reaction). (**b**) Panel for *E. coli*: TSI(A/A), 2: urease (negative reaction), 3: citrate utilization test (negative reaction), 4: peptone water for Indole (positive reaction), 5: methyl red test (positive reaction).

The bacteria that grew were further characterized basing on their gram-staining properties and biochemical traits, under the guidance of the Bergey’s Manual of Systematic Bacteriology^21^ (Fig. 5, Table 5).

**Table 5:** Gram staining properties, cellular morphology, and biochemical traits of the representative (a) gram-negative, and (b) gram-positive bacteria isolated from commercial herbal medicines in Kampala city.

(a) Gram-negative bacteria											
Isolate ID. No	Bacterial species isolated	Gram reaction, shape	Biochemical characteristics								
			TSI			H_2_S	Citrate	Urease	Indole	MR	VP
			B	S	G						
A40	*K. pneumoniae*	G−, Rods	A	A	+	−	+	+	−	−	+
A09	*K. pneumoniae*	G−, Rods	A	A	+	−	+	+	−	−	+
A18	*P. aeruginosa*	G−, Rods	NC	K	−	−	+	+	−	−	+
A58	*K. pneumoniae*	G−, Rods	A	A	−	−	+	+	−	−	+
A64	*K. oxytoca*	G−, Rods	A	A	−	−	+	+	+	−	+
A55	*K. pneumoniae*	G−, Rods	A	A	−	−	+	+	−	−	+
A139	*K. oxytoca*	G−, Rods	A	A	−	−	+	+	+	−	+
A55	*K. oxytoca*	G−, Rods	A	A	−	−	+	+	+	−	+
A33	*E. coli*	G−, Rods	A	A	−	−	−	−	+	+	−
A65	*E. coli*	G−, Rods	A	A	−	−	−	−	+	+	−
A15	*Enterobacter* spp.	G−, Rods	A	A	−	−	+	+	−	−	+

(b) Gram-positive bacteria											
Isolate ID. No	Gram reaction, shape	Bacterial species isolated	Biochemical characteristics								
			Mannitol fermentation	Coagulase	Catalase	DNase					
A23	G+ cocci arranged in grape-like clusters	*S. aureus*	+	+	+	+					
A42	G+ cocci arranged in grape-like clusters	*S. aureus*	+	+	+	+					

*ID. No* identification number, *VP* Voges–Proskauer, *MR* methyl red, *H*_*2*_*S* hydrogen sulfide, *B* Butt, *S* Slant, *G* gas, *K* red/alkaline, *NC* no colour change, *TSI* triple sugar iron, *G−* gram-negative, *G+* gram-positive, +  positive,− negative, *E. coli* *Escherichia coli*, *K. pneumoniae* *Klebsiella pneumoniae*, *K. oxytoca* *Klebsiella oxytoca*, *S.* *aureus* *Staphylococcus aureus*, *P. aeruginosa* *Pseudomonas aeruginosa*, *DNase* Deoxyribonuclease.

### Bacterial species isolated from selected herbal medicines sold in Kampala city

A total of 59 bacterial strains were recovered from 45 (32.1%), of the 140 HM samples screened. The majority were *K. pneumoniae* (34; 57.6%), followed by *E. coli* (12; 20.3%), while the minority were *Salmonella* spp. (0; 0.0%) and *Shigella* spp. (0; 0.0%) (Table 6). The microbes were isolated from 35 out of 115 liquid samples (30.4%), with the exception of 11 isolates, *viz*; *K. pneumoniae* (5; 8.5%), *E. coli* (3; 5.1%), *S. aureus* (2; 3.4%), and *K. oxytoca* (1; 1.7%), which were got from 10 (40.0%), out of the 25 solids screened.

**Table 6:** Bacterial strains isolated from herbal medicines sold in five divisions of Kampala city (n = 59).

Bacterial species	Number of bacteria isolated					Total (N; %)
	Kawempe	Central	Nakawa	Makindye	Rubaga	
*Klebsiella pneumoniae*	10	13	5	6	0	34; 57.6
*Escherichia coli*	4	3	4	1	0	12; 20.3
*Staphylococcus aureus*	3	3	0	0	1	7; 11.9
*Klebsiella oxytoca*	1	1	1	0	0	3; 5.1
*Bacillus cereus*	1	0	0	0	0	1; 1.7
*Pseudomonas aeruginosa*	1	0	0	0	0	1; 1.7
*Enterobacter* spp.	1	0	0	0	0	1; 1.7
*Salmonella* spp.	0	0	0	0	0	0; 0.0
*Shigella* spp.	0	0	0	0	0	0; 0.0
Total (N; %)	21; 35.6	20; 33.9	10; 16.9	7; 11.9	1; 1.7	**59; 100**

## Discussion

### Socio-demographic profiles of participants

The majority of the participants recruited in this study were men. The predominance of men in HM trade was reported earlier in some parts South Africa^22^, Tanzania^23^, and Malawi^24^. However, in some provinces of South Africa such as KwaZulu-Natal, and Mpumalanga^25^, the majority of commercial herbalists were reported to be women, and this aligned with findings of most studies that were conducted in the rural herbalist communities in several parts of Uganda^26–29^. Though men’s predominance as commercial herbalists in urban settings, as opposed to the rural areas, has been well documented, the factors that underpin this gender disproportion remain largely unexplained. Partly, this gender disparity may be attributed to societal norms^28^, and also to the fact that females are commonly responsible for their families’ primary health care. Hence treating people becomes a responsibility of mainly the female gender most evidently in the rural settings^28^.

Our observation, that over 90% of the participants in the current study had attained a certain level of formal education is of great significance, since basic education is believed to be a critical component of the health^30^. The high profits gained from HM sales, as observed in this study, highlight the potential role herbal medicines play towards Uganda’s national economy, as well as supporting the attainment of community livelihood needs, primary health care, and cultural heritage. Consequently, medicinal plants have been acknowledged as an important feature of the cultural, economic, medicinal, and ecological components of all cities in the world^31^.

### Characteristics of the herbal medicines from which bacterial contaminants were isolated

Both the solid and liquid HM were included in this study and were procured from herbal shops, market stalls, as well as roadside and/or mobile stalls. Likewise, such types of commercial HM establishments were reported in urban settings in other parts of Africa and beyond^6,32–35^. The abundant presence of roadside and/or mobile commercial herbal medicines might pose herbal quality challenges since adequate regulation and monitoring of such establishments can be complicated^36^. Also, our observation that some HM were placed in recycled packaging materials (previously discarded as wastes) points to further potential threats to herbal safety since the re-use of these packages has been linked to the introduction of pathogenic microbial contaminants into the drugs elsewhere^33,34,36,37^. Even though the proportion of HM that absolutely violated the Uganda National Drug Authority (NDA)’s herbal packaging and labeling guidelines was generally high, it demonstrated a twofold decrease in the percentage that was reported in Makindye division of Kampala over a decade ago^5^. The NDA-guidelines provide for ten conditions that should be fulfilled to attain the desired herbal packaging and labelling standards, viz; (1) tight packaging (absence of leakage), (2) presence of a brand name, (3) active ingredients (contents), (4) indications (diseases/therapeutic claims), and contraindications (warnings and precautions), (5) name of manufacturer, (6) full address of manufacturer with physical location, (7) batch number, (8) date of manufacture and expiry, (9) dosage (for adults and children), and (10) directions for use and storage^38^. The factors that could explain this improvement in conformity of the HM sold in Kampala to the NDA-guidelines remain unclear. However, it might potentially be partly attributed to the rising competition which has stimulated innovations not only in the marketing/advertisement but also improvement in packaging and branding, nearly comparable to the standards that are acceptable in pharmacies^39^. Nonetheless, some hinderances to the standard labelling of herbal medicines have been reported in some parts of the world; for example, most herbalists decline to specify the active ingredients/contents present in their herbal products because they wish to keep their traditional medicinal knowledge confidential^5,40^. Unfortunately, this may lead to in adverse drug reactions if members of the community use HM having ingredients to which they are allergic.

Much as our inclusion criteria sought a random sample of 140 herbal medicines used for cough, diabetes, Ulcers, and/or diarrhea treatment, the same remedies were found to be used in the management of 62 health complications, including HIV/AIDS, different types of cancers, and reproductive disorders among others. On average therefore, each remedy was suggested to treat over 15 ailments. Though the “one drug for many diseases” approach may not only reduce the cost of treatment of multiple infections^41^, it can also increase the number of individuals (suffering from different ailments) who may consume the same drug, and hence get exposed to its associated risks such as pathogenic microbial contaminants.

### Total viable loads (TVL) of bacteria isolated from selected herbal medicines sold in Kampala City

According to the quality control guidelines set by World Health Organization, herbal medicines with total viable loads of bacteria that exceed the maximum permissible limits (10^5^ CFU/ml for liquids and 10^7^ CFU/g for solids) should be considered unsuitable for human consumption and hence rejected^18,19^. The guidelines provided by other establishments such as the European pharmacopeia generally align with the standards set by the WHO^42,43^. In the current study, 35.7% of the HM possessed bacterial TVL that exceeded the maximum acceptable levels in reference to the WHO standards^18,19^. Even though most of these unsuitable samples were found in Rubaga division, the mounts did not significantly differ from those found in the other parts of the city (*χ*^2^, *p* < 0.05). The proportion of HM which exceeded the permissible TVL of bacteria in Kampala that were revealed in this study is over threefold higher than that (11.7%), reported by another study in 2009^5^. The difference might partly be explained by the fact that the latter^5^, was less comprehensive since the sampling was limited to liquid HM for cough in Makindye division alone. Other few previous studies that assessed the bacterial contamination of herbal medicines in Kampala did not examine this parameter (the total viable loads of bacteria)^14,44,45^.

Our findings revealed that HM with ≤ 50% level of conformity to Uganda National Drug Authority’s packaging and labeling guidelines contained significantly higher TVL values than those with > 50%, and that there were no significant differences in the contamination levels between the highly priced and the low-cost HM. This implies that irrespective of the price, some of the commercial HM in Kampala may disseminate viable bacteria in the community, but conformity to the Uganda National Drug Authority guidelines can play a considerable role in resolving this burden. Most bacteria have the potential not only to impair human health but also spoil and reduce the potency of the herbal medicines they contaminate^46^.

### Total Coliform Counts (TCC), and *E. coli* counts

In the present study, the proportion of HM (42.1%), which were found to exceed the permissible level of total coliform counts was high give the perceived public health importance of most coliform bacteria^5,46,47^. The term “total coliforms” represents a group of non-spore-forming, gram-negative, facultatively anaerobic, bacillus bacteria that strongly ferment lactose to acid and gas following 42–48 h incubation at 35 ± 2 °C^48^. They mainly belong to two groups. The 1st group includes bacteria that often form part of the natural flora of most vegetation, such as some strains of *Enterobacter* spp., Serratia spp., and *Erwinia* spp. among others. The bacteria belonging to this group do not have any historicaly linkages to feces and are commonly prevalent in environmental reservoirs such as water and soil. The presence of these strains in consumable products like HM may not necessarily infer fecal contamination, hence they do not necessarily indicate a substantial public health threat^49^. The 2nd group constitutes thermotolerant (fecal) coliforms, which are commonly found in the intestines of man and other warm-blooded animals^48^. These include some strains of *Escherichia* spp, *Klebsiella* spp, and *Citrobacter* spp. among others. The strains belonging to this group largely constitute the intestinal flora of man and other warm-blooded animals, hence they signify potential contamination by fecal matter and its associated human pathogens, threatening public health^16^. *Escherichia coli* is believed to be one of commonest flora that inhabit the intestinal tract of vertebrates^50^. Therefore, the presence of this coliform is widely used as a reliable basis to infer the degree of fecal contamination.

In the current study, over eight in very one hundred herbal medicines were found to possess higher loads of *E. coli* than the permissible level (10 CFU/ml or g), as per the guidelines of WHO^18^. From the public health point of view, over 8% of the samples screened in the current study were therefore unsafe for human use on the basis of this parameter. A chi-square test revealed that the overall number of samples with unacceptable total coliform counts were significantly higher than those harboring undesirable *E. coli* loads (*p* = 0.0291). Consequently, it was surprising to note that Rubaga division did not possess any samples with unacceptable levels of *E. coli* even though over 35% harbored high total coliform counts. These observations demonstrate the potential role other coliform bacteria (most likely nonfecal coliforms that are commonly prevalent on plants, and in environmental resources like soil and water), play in contaminating herbal remedies, hence the need to observe both the good manufacturing practices as well as the good agricultural practices by the herbalists.

### Specific bacterial strains isolated from selected herbal medicines sold in Kampala city

**Figure 6:**
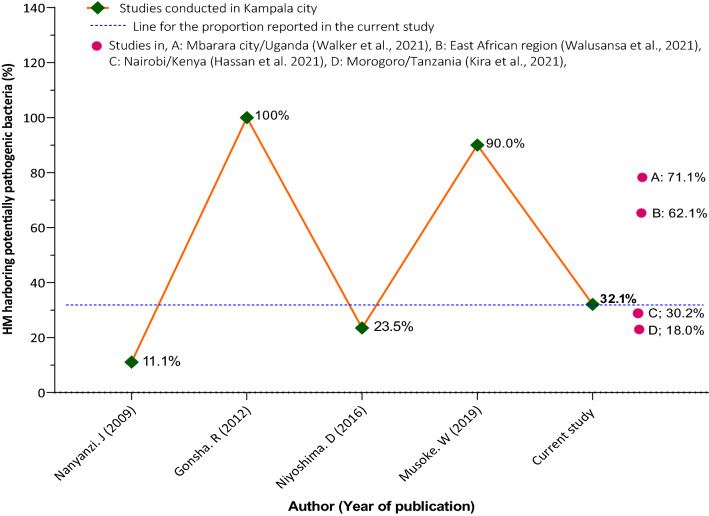
% of herbal medicines (HM) reported to be contaminated with potentially pathogenic bacteria in Kampala and its neighborhoods^1,5,6,14,17,44,45,51^.

We report a 32.1% proportion of HM that were contaminated with at least one bacterial species of potential medical importance. Since the beginning of the 21st century, the percentages of bacterially contaminated HM in Kampala city have demonstrated an oscillating pattern, with some studies revealing less and others higher values than the 32.1% as well as the 62.1% reported by the current study and the East African regionwide survey respectively (Fig. 6)^1,5,14,44^. This creates uncertainties about the likely trend which the HM bacterial contamination burden in Kampala might ensue subsequent to the present study.

The failure to attain a stable level of microbiological safety in the herbal medicines sold in Kampala city, and/or some other similar settings (Fig. 4), reflects intervention gaps which could be associated with the policy and/or regulatory environment, hygiene, and sociographic factors of herbalists among others. In the present study, it was outrageous to observe the concurrence of three bacterial strains of potential medical importance in two of the herbal liquids (packed in dusty, re-used mineral water bottles), one for cough and the other for diarrhea treatment. The former was grossly adulterated with *P. aeruginosa* and the latter harbored *S. aureus*, while both were positive for *E. coli* and *K. pneumoniae*. Such HM might promote the community spread of mixed bacterial infections which can be complicated to treat. It was commendable to note that in this study, many of the primary pathogens such as *Salmonella* spp. and *Shigella* spp., some of which have been associated with persistent outbreaks of diarrheal diseases like typhoid fever in Kampala city were absent in all the samples screened^15^. Nonetheless, the strains that have been revealed by the current study to be highly prevalent in HM in Kampala, particularly *K. pneumoniae, E. coli* and *S. aureus* have the potential to impair human health in several ways. For example, *S. aureus*, and several pathotypes of *E. coli* are involved in severe diarrhea and/or urinogenital infections^16,52,53^. Besides, these pathogens are on the global list of critical multi-drug-resistant bacteria which require urgent redress^54^. Although bacterial contaminants have been reported in some conventional pharmaceutical drugs elsewhere^55^, the modern drugs included as controls in the present study where safe in reference to all the microbiological parameters examined. This implies that the modern drugs sold in pharmacies in Kampala might be of suitable microbiological quality but this can only be confirmed following a comprehensive investigation.

## Conclusions

The prevalence of commercial herbal medicines that are bacterially contaminated in Kampala city remains relatively high. Some strains belonging to the bacterial species involved in this contamination, particularly, *E. coli*, *K. pneumoniae, P. aeruginosa* and *S. aureus* have the potential to cause severe clinical diseases, spoilage of HM, and deterrence of therapeutic potency. Also, these strains have been implicated in the community spread of antibiotic resistance. There is need to conduct further research on bacteria isolated from HM in Uganda, especially to examine their antibiotic resistance traits, to fully elucidate the risk incurred. Quality assurance should be enhanced to promote public health and to harness herbal medicine sector development.

## Methods

### Study area

**Figure 7:**
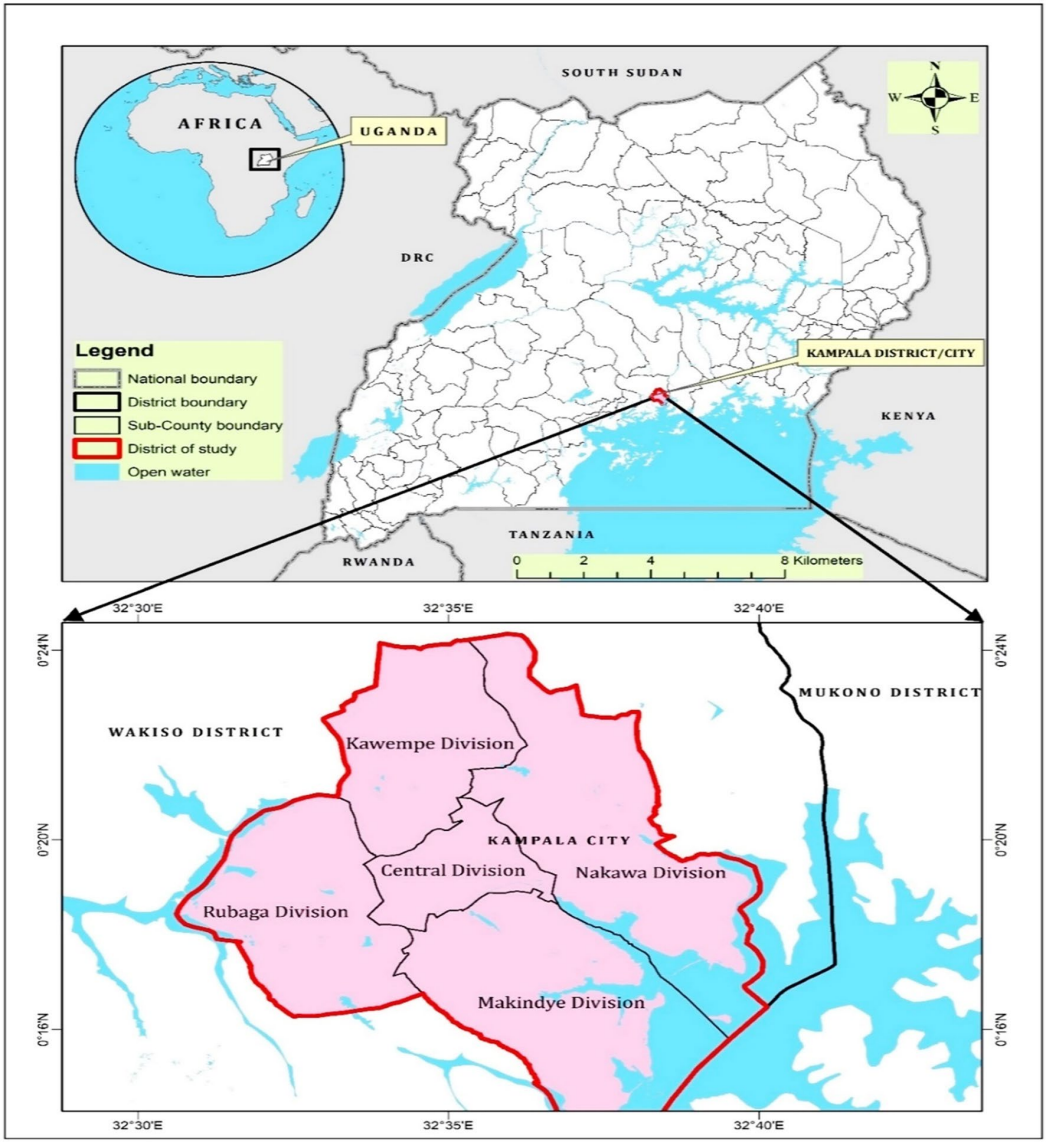
Study locale: Kampala city showing the five administrative divisions.

The study was conducted in the five administrative divisions of Kampala city, located in the central region of Uganda, stretching over an area between DMS Latitude: 0° 12′ 46.755″ N, Longitude: 32° 30′ 32.567″ E and DMS Latitude: 0° 12′ 20.692″ N, Longitude: 32° 40′ 14.054″ E. It is bordered by Mukono district to the East; Lake Victoria to the South East; and Wakiso district to the West, South, and North (Fig. 7). Its five administrative divisions are; Kampala central, Kawempe, Nakawa, Makindye and Rubaga divisions. According to the recent Uganda national population census, the city is populated by about 1,680,601–2,915,200 residential occupants^56^, plus large numbers of individuals that enter and leave the city on a daily basis^11^.

### Study design

A cross sectional survey was conducted on 65 traditional herbalists that sell herbal medicines in Kampala, between April and September 2021. Pre-tested, semi-structured questionnaires were used to record the socio-demographic profiles of respondents, and the attributes of herbal medicines which were procured from the participants. Such attributes included, prices, quantity and label information among others. In addition, HM packaging properties, categories of traditional HM outlets, cleanliness, and pharmaceutical forms of the HM were examined through field observations which were supplemented using photography, with a high-resolution digital camera inbuilt in a Phantom-9 Mobile Phone, model AB7/2019, Techno Mobile Limited^57^. The laboratory experiments were aimed at assessing the Total Viable Loads (TVL), Total Coliform Counts (TCC), as well as the isolation and characterization of selected diarrheal and/or respiratory bacterial pathogens from the herbal medicine samples. The targeted bacterial species were those that had been commonly implicated in disease causation in Kampala city and/or Uganda at large, such as; *Salmonella* spp., *E. coli* and *K. pneumoniae* among others^15,16,58^.

### Study population

The study units of interest in this research were the herbal medicines for diarrhea, cough, diabetes, and high blood pressure, sold by herbalists in herbal shops, market stalls, and roadside/mobile stalls in Kampala city. These herbal medicines were selected because of their perceived high frequency of use, and the likely carriage of bacterial contaminants of mostly unidentified profiles^5,14^.

### Sample size

Due to high number of largely unregulated HM marketed with various therapeutic claims in Kampala, a representative sample (N), was determined using the formula for unknown population size at 95% confidence interval; N = Z^2^_1−α_ [P (1 − P)] ÷ D^2^^59^.

Where; N = The desired sample size, Z_1−α_ = Standard error of the mean = 1.96 at 95% confidence level, P = Estimated prevalence of bacterial contamination in HM above the WHO permissible levels = 90%^14^, and D = Tolerable sampling error = 0.05 at 5% significance level.

Hence, sample size = 1.96^2^ [0.9 (1 − 0.9)] ÷ 0.05^2^ = 138.3 ≃ 138.

Therefore, the minimum sample size required was 138. However, since the current study was conducted in five administrative divisions of Kampala, a sample size of 140 herbal medicines was adopted, and was divided into strata (of 28 samples each), that were obtained from each division. In addition, two conventional pharmaceutical drugs (one powder for diarrhea, and one liquid for cough), from pharmacies were included as controls; hence the total of medicines processed for bacteriological analysis was 142.

### Sampling technique

Prior informed consent was obtained from all the herbalists that offered their herbal medicine samples to be used in this study. Ethical approval for the study was obtained from the School of Health Sciences Research and Ethics Committee of Makerere University. The sampling frame exclusively involved herbalists that were engaged not only in trade, but also in harvesting, and/or preparation of the HM. The stratified sampling technique was adopted; viz, each administrative division of Kampala constituted a stratum 28 samples which were procured from herbal shops, market stalls, roadside stalls, and mobile stalls. Then, the random selection of individual herbal medicine samples from each stratum was done following the guidelines for research on HM products, established by the Uganda National Drug Authority (NDA) and the World Health organization^38,60^.

### Specimen collection and transportation

We randomly selected some of the HM (ready for consumption), that had been proposed to potentially possess largely unknown species of bacterial contaminants in earlier studies, particularly those for diarrhea, cough, diabetes, and high blood pressure^5,14^. Upon procurement, the samples were aseptically wrapped in sterile zipped specimen bags, and transported on a cold chain for bacteriological analysis, in the microbiology laboratory at Makerere University, School of Veterinary Medicine. Since microbial loads may depend on temperature and humidity^47^; the samples were collected during dryness as well as frequent rains, from April to September 2021.

### Media preparation, and bacteriological analysis

Three types of bacteriological analyses were performed, viz: (1) determination of bacterial total viable counts and total coliform counts, and (2) determination of total *Escherichia coli* counts, and (3) isolation and biochemical characterization of *Klebsiella pneumoniae*, *Escherichia coli*, *Klebsiella oxytoca*, *Pseudomonas aeruginosa, Enterobacter* spp.*, Staphylococcus aureus, Salmonella* spp., *Shigella* spp., and *Bacillus cereus*.

#### Media preparation

All the media used in this study were prepared and casted into sterile bacterial culture plates by following the manufactures instructions. The bacterial total viable count was determined using Total Plate Count Agar (PCA), Batch Number 805161; while total coliform count was determined using Violet Red Bile Agar (VRBA), Batch Number: 605181. Both media were procured from Conda Pronadisa (Laboratorion Conda S.A.), in Spain (www.condalab.com). *Pseudomonas aeruginosa* was isolated using Kings Agar medium (KMA); Lot Number: 213075, manufactured by Techno Phermchem, Bahadurgarh, India. *Staphylococcus aureus* was isolated using Mannitol Salt Agar (MSA); Lot Number: 3213811, procured from Oxoid Ltd, Basingstoke, United Kingdom. *Bacillus cereus* was isolated using *Bacillus cereus* agar (BCA), Lot Number: 0000132719, manufactured by HiMedia Laboratories Pvt. Ltd, Mumbai, India. *Salmonella* spp. and *Shigella* spp. were isolated using Xylose Lysine Deoxycholate (XLD) agar; Lot Number: 2875014, manufactured by Oxoid Ltd, United Kingdom. Prior to culturing on XLD, the samples were first pre-enriched in peptone water; Batch Number: 602041, manufactured by Conda Pronadisa, Spain, followed by enrichment in Rappaport-Vassiliadis Soya Peptone (RVS) broth; Lot number: 2896635, manufactured by Oxoid Ltd, United Kingdom. All other coliform bacteria, such as *E. coli*, *Klebsiella* spp., and *Enterobacter* spp. were recovered from the colonies that grew on VRBA, during determination of TCC. Screening for Enterohaemorrhagic *E. coli* (017H7) was done using Sorbitol MacConkey; Lot number; 461684 procured from Oxoid Ltd, Basingstoke, United Kingdom. All the media was quality controlled through overnight incubation at 37 °C prior to inoculation, to rule out contamination.

#### Preparation of serial dilutions of herbal medicine samples

For the liquid samples (10° dilution), five test tubes (labeled 1–5) were arranged and 9.0 mls of sterile peptone water introduced into each tube. Then 1.0 ml of a sample was pipetted into test tube number 1 followed by thorough mixing, hence constituting the 10^–1^ dilution. 1.0 ml of the 10^–1^ dilution (in test tube 1), was pipetted into test tube 2 using a fresh pipette tip, followed by thorough mixing, hence constituting the 10^–2^ dilution, and the procedure was repeated up to test tube number 5, thus constituting the dilutions 10^–3^, 10^–4^ and 10^–5^, respectively. For the case of solid samples, 10 g of each sample were introduced into a stomach bag containing 90 mls of peptone water. The mixture was homogenized using a Pulsifier (Serial No: 30660282, Manufactured by Kalyx Biosciences Inc, Canada), and this constituted the 10^0^ dilution which was then serially diluted up to the 10^–5^ through the same procedure applied above on liquid samples.

#### Determination of total viable counts (TVC)

0.1 ml of each serial dilution of the herbal medicine samples were pipetted onto PCA media plates, spread using a glass spreader, and incubated for 24 h at 37 °C. Colonies that grew were counted using a colony counter (CAT No: SC6+, Serial number: R570003270, made in United Kingdom, www.staurt.equipment.com), and expressed as Colony Forming Units per gram (CFU/g) for solid samples, or per milliliter (CFU/ml) for liquids. To convert the counted colonies into CFU, a standard formula (below), was used^61^.

$$CFU=\frac{Number \, of \, Colonies \, Counted }{(Dilution \, Factor \times Volume of \, Sample \, plated)}$$

The CFU of the different dilutions were computed and the mean was evaluated and recorded as the TVC of that sample. The mean TVC for each sample was compared with the permissible limits recommended by the World Health Organization^18,19^, to assess whether the samples were of good quality and safe for human consumption.

#### Determination of Total Coliform count (TCC)

0.1 ml of each dilution was pipetted onto VRBA media plates, spread using a glass spreader, and incubated for 24 h at 37 °C. Colonies that grew were counted using a colony counter (CAT No: SC6+, Serial number: R570003270, made in United Kingdom, www.staurt.equipment.com) and expressed as Colony Forming Units per gram (CFU/g) for solid samples, or per milliliter (CFU/ml) for liquids. The coliforms were considered as pale pink, moist/mucoid colonies that grew on VRBA media, except for *E. coli* which presented as hot pink, dry, flat/umbonate colonies. The CFU of the different dilutions were computed and the mean was evaluated and recorded as the TCC of the sample. The mean TCC for each sample was compared with the permissible limits recommended by the World Health Organization^18^, to assess whether the samples were of good quality and safe for human consumption. All samples with a mean TCC > 0.0 CFU/ml, or > 0.0 CFU/g were classified as unsafe for human consumption (WHO, 2007).

#### Isolation and identification of the targeted bacteria *P. aeruginosa*, *S. aureus*, *B. cereus*, *E. coli*, *Klebsiella *spp., *Enterobacter *spp., *Salmonella* spp. and *Shigella* spp.

To isolate the targeted bacterial species, 0.1 ml of the 10^–1^ dilution of each sample was spread plated on the appropriate culture media, viz; *Pseudomonas aeruginosa* (on Kings agar), *Staphylococcus aureus* (MSA), *Bacillus cereus* (BCA), *E. coli*, *Klebsiella* spp., and *Enterobacter* spp. (VRBA). For the case of *Salmonella* spp. and *Shigella* spp., prior to culturing on XLD agar, the samples were first taken through a pre-enrichment stage, followed by an enrichment process. Pre-enrichment was done by incubating the 10^–1^ dilution of each sample at 37 °C for 24 h. The enrichment culture procedure was then done by introducing 1.0 ml of the 24-h old pre-enriched culture into 9.0 mls of Rappaport–Vassiliadis Soya Peptone broth, followed by incubated for 24 h at 42 °C. One loopful of the enriched cultures were then streak plated on XLD.

All the inoculated agar plates were incubated at 37°C for 24 h. In the case of *E. coli*, the pink colonies that grew on VRBA were sub-cultured on sorbitol MacConkey and incubated at 37 °C for 24 h to screen for the potential presence of translucent colonies of Enterohaemorrhagic *E. coli* (*E. coli*: 017H7). The observed bacterial species in each sample were compared with the guidelines of the World Health Organization to assess if the samples were safe for human consumption (WHO^10,18^).

The bacterial colonies that grow were identified based on their cultural characteristics (growth and colony morphotypes on various forms of media), gram staining, and biochemical traits^62^. The biochemical experiments used for bacterial characterization in this study were as follows;

- The catalase test: The catalase enzyme catalyzes the dissociation of hydrogen peroxide (a cytotoxic metabolite) to nontoxic products (water and oxygen), i.e., ${\text{2H}}_{{\text{2}}} {\text{O}}_{{\text{2}}} \left( {\text{l}} \right) \to {\text{2H}}_{{\text{2}}} {\text{O }}\left( {\text{l}} \right) + {\text{O}}_{{\text{2}}} \left( {\text{g}} \right)$. A loopful of 24 h-old bacterial colonies was introduced onto the inner walls of a screw-cap test tube containing 0.5 ml of H_2_O_2_ solution. The test tube was then capped (to prevent the escape of aerosols), and slanted in order for the H_2_O_2_ solution cover the bacterial colonies. Effervescence in about 30 s was indicative of a positive reaction^63^.
- Urease test: The urease test examines the ability of the bacterial species to produce urease enzyme. This enzyme catalyzes the hydrolysis of urea into ammonia and carbondioxide, i.e. ${\text{(NH}}_{{\text{2}}} )_{{\text{2}}} {\text{CO}}\left( {{\text{aq}}} \right)_{~} + {\text{ H}}_{{\text{2}}} {\text{O}}\left( {\text{l}} \right) \to {\text{2NH}}_{{\text{3}}} \left( {\text{g}} \right)~~ + {\text{CO}}_{{\text{2}}} \left( {\text{g}} \right).$. The ammonia formed ionizes, resulting in an alkaline medium, *viz*; ${\text{NH}}_{{\text{3}}} \left( {\text{g}} \right)~~ + {\text{H}}_{{\text{2}}} {\text{O}}\left( {\text{l}} \right)~ \to {\text{NH}}_{{\text{4}}} ^{ + } \left( {{\text{aq}}} \right)~~ + {\text{ OH}}^{ - } \left( {{\text{aq}}} \right)$. The alkalinity is detected using a suitable Ph-indicator^63,64^. The butt and surface of a slant of 3.0 ml of urea agar base (containing phenol red indicator) supplemented with 5% v/v of 40% w/v urea solution, in a screw-cap test tube, was inoculated with 24 h-old bacterial colonies, and incubated at 37 °C for 24 h. The development of a red color in the urea agar was indicative of a positive urease reaction.
- Indole production/tryptophan decomposition test: This test demonstrates the presence of tryptophanase enzyme in bacterial isolates, which enables them to metabolize the amino acid tryptophan. This enzyme catalyzes the breakdown of l-tryptophan into indole, pyruvic acid and ammonia. The indole is detected when it reacts with p-dimethylaminobenzaldehyde to form a quinoidal red-violet (red or pink) compound^65^. 2.0 mls of peptone broth in a screw-cap test tube were inoculated with a loopful of 24-h old bacterial colonies and incubated at 37 °C for 24 h. Then 3 drops of Kovac’s reagent were added to this broth culture. Formation of a red or pink ring on top of the broth culture was indicative of indole positive isolates.
- Methyl red (MR) test: Both the MR test and the Voges–Proskauer (V–P) test are used to identify the fermentation pathways of bacterial isolates for acids and carbohydrates, respectively^66^. The MR test examines the ability of bacterial strains to catabolize glucose in a buffered media hence yielding ethanol, CO_2_, H_2_, and a mixture of organic acids such as succinic acid, lactic acid, formic acid, and acetic acid. The acids cause a drop in the pH of the culture below 4.2, and this is detected using a suitable indicator^65,66^. 2.0 mls of sterile methyl red-Voges–Proskauer (V–P) medium (glucose-phosphate) base in screw-cap test tubes were inoculated with a loopful of 24-h old bacterial colonies and incubated at 37 °C for 4 days, and then a drop of methyl red reagent (0.25 g of methyl red in 100 mls of ethanol) was added. The results were interpreted basing on the emergent colour changes, i.e., red colour (MR positive), any shade of orange or yellow was taken as a negative reaction.
- V–P test (Voges–Proskauer test): This test is used to identify fermentative microbes that catabolize glucose via the butanediol pathway. During the glucose fermentation, acetoin (acetylmethylcarbinol) exists as an intermediate in the formation of 2,3-butanediol, which gets oxidized (in the presence of KOH and O_2_) to a diacetyl. The diacetyl reacts with a guanidine group present in amino acids such as arginine (contributed by peptone in the V–P media), to form a pink- to red-colored product and this colour may be intensified by α-naphthol^63,65^. 1.0 ml of MR-VP reagent in a screw-cap test tubes were inoculated with a loopful of 24-h old bacterial colonies and incubated at 37 °C for 4 days. 3 drops of reagent A (5.0 g of α-naphthol in 100 mls of absolute ethanol) were added and followed by 3 drops B (40.0 g of KOH in 100 mls of distilled water). Development of strong cherry red color on the surface of the media was indicative of a positive V–P test while a copper or lack of colouration showed a negative result.
- Hydrogen sulfide (H_2_S) test: This examines the ability of microbes to produce hydrogen sulfide (H_2_S), through the anaerobic reduction of thiosulfate molecules (S_2_O_3_^2−^). The detection of H_2_S is achieved through its reaction with Iron salts (present in the culture media), which yields black precipitates of either Iron(ii) Sulfide (FeS) or Iron(iii) Sulfides (Fe_2_S_3_)^63,67^. Using a stabbing wire, a 24-h old culture was stabbed into SIM (Sulfite Indole Motility) media in screw-cap test tubes and incubated at 37 °C for 24 h. The formation of black coloration in the media indicated positive results. This same test was used to assess the motility and indole production. Motile organisms were identified based on their observable growth away from the stabbing line, while indole production was deduced by adding a drop of Kovac’s reagent and observing the formation of pink colouration.
- Triple Sugar Iron test (TSI): This test identifies bacterial strains basing on their differences in the carbohydrate fermentation patterns and hydrogen sulfide production ability^66^. The TSI agar contains sodium thiosulfate, peptone, three fermentative carbohydrates [lactose (1%), sucrose (1%) and glucose (0.1%)], and the phenol red acid base indicator. The accumulation of acids associated with carbohydrate fermentation, lowers the pH hance the phenol red indicator turns from orange-red to yellow, while the oxidative decarboxylation of peptone yields alkaline products hence the indicator turns deep red. The production of H_2_S is indicated by the black colour of the butt^66^. The TSI agar in the screw-cap test tubes was inoculated with 24-h old bacterial colonies, first by stabbing through the center of the media up to the bottom of the butt, followed by streaking on the surface of the slant. The tube was loosely capped and incubated at 37 °C for 24 h. Alkaline/acid (red slant/yellow butt) was interpreted as glucose fermentation only; acid/acid (yellow slant/yellow butt) as metabolization of glucose, lactose and/or sucrose; alkaline/alkaline (red slant/red butt) as absence of any carbohydrate fermentation; blackening of the media as H_2_S production, while bubbles or cracks in the agar were interpreted as gas (CO_2_ and H_2_) production.

#### Data analysis

Data were entered in MS-Excel and exported to STATA version 15.0 software. The data were tested for normality by using D'Agostino–Pearson test and normal distribution was accepted at (*p *> 0.05). Data were then analyzed using descriptive statistics (frequencies and percentages), and inferential statistics [Pearson’s Chi-square (in case of normality), or Mann–Whitney U test (for nonparametric data)], at 0.05 significancy level.

### Ethical approval and consent to participate

The study sought ethical approval from the Makerere University School of Health Sciences Research Ethics Committee (Ref: MAKSHSREC-2020-72), Uganda National Council for Science and Technology (Ref: HS1278ES), and Kampala Capital City Authority (Ref: DPHE/KCCA/1301). The research was conducted in conformity to the national guidelines for the conduct of research in the COVID-19 era established by the Uganda National Council for Science and Technology (UNCST)^68^. Informed consent to participate in this study was obtained in writing from the study participants. Respondents’ identifiers were recorded in form of assigned codes instead of names to ensure anonymity.

## Data Availability

All data generated and analyzed during this study are available on request from the corresponding author.

## Abbreviations

HM: Herbal medicine
TMP: Traditional Medical Practitioners
WHO: World Health Organization
KCCA: Kampala Capital City Authority
